# Measuring the impact of structural inequality on the structure of the brain

**DOI:** 10.1073/pnas.2306076120

**Published:** 2023-06-07

**Authors:** Margaret A. Sheridan

**Affiliations:** ^a^Department of Psychology and Neuroscience, University of North Carolina, Chapel Hill, NC 27599

In their recent paper, Zugman et al. (1) have made significant trides in capturing the relationship between gender inequality and brain structure through a cross-country comparison. The authors explore the association between gender inequality at the country level and gender differences in neural structure at the individual level. The analysis is an excellent use of similar neural data acquired across a variety of country settings. This paper represents a truly interesting step forward in work examining the biologic embedding of structural inequality, while also serving as an excellent example of the value of collaborative and open science initiatives.

In 2010, Hertzman and Boyce proposed that social inequality could “get under the skin” impacting long-term health and well-being (2). This concept, known as biologic embedding, is one mechanism through which social inequality leads to differences in health outcomes. Biologic embedding has shaped explanatory models for the association between socioeconomic status, race, and, to a lesser degree, gender, and health outcomes.

Biologic embedding has been proposed as one explanation for the observation that across country settings and time, strong associations have been observed between socioeconomic status and almost every health outcome, including all-cause morbidity and mortality (3). In cross-country comparisons, researchers have observed that wealthy countries with more economically equal social hierarchies (lower Gini coefficients) have less strong social gradients of health outcomes than those, like the United States with relatively unmitigated social gradients (4). These gradients in health, observed at the aggregate level of the population, are almost certainly explained by a wide number of factors. Economic resources confer direct access to purchasing health care even in societies with socialized medicine. In addition, economic resources can be used to increase leisure, access social networks which support well-being, and purchase education which can be used to address health deficits. The concept of biologic embedding is in addition to these resource-based explanations of the impact of social hierarchy on health. Within the concept of biologic embedding, two primary possibilities have been explored: first, the possibility that social inequality aggregates exposure to adversities which directly impact biologic development, and second, that the experience of inequality itself can shape biology and thus health outcomes.

Evidence for both possibilities is extensive and has been studied in relatively separate, albeit intersecting pieces of literature. A vast body of work has demonstrated that experiences of adversity such as direct exposure to interpersonal violence or lack of parental care, particularly when experienced in early childhood, have long-term impacts on brain development (5). Recent randomized studies indicate that long-term changes in brain structure are caused by early social experiences and that these are associated with long-term changes in mental health outcomes (Sheridan et al., 2022). Other studies show that specific experiences of social inequality, that is being lower in a social hierarchy, cause short-term changes in hypothalamic–pituitary adrenal (HPA) axis, immune, and brain function. Numerous association studies indicate that when these experiences aggregate across time, allostatic load increases, negatively impacting health (6). Studies documenting these effects in humans are bolstered by a large body of preclinical studies which have identified the mechanisms through which early exposure to adversities and low social status comes to impact gene expression, immune, HPA axis function, and brain development (7).

In many of these studies, adversity or low social status is either self-reported by individuals or randomly assigned to participants by nature or experiments. While these studies have provided valuable insights into the mechanisms and pathways through which social inequality affects neurodevelopment, they are unable to fully identify the impact of structural inequality. Research in race and gender-based inequality has demonstrated that measurement of direct experiences of interpersonal racism or sexism only identifies a fraction of the pathways through which social inequality impacts health (8). A direct experience of sexism (e.g., a sexist remark) may impact an individual’s well-being momentarily or when experienced iteratively over time could lead to increases in allostatic load. However, most of the power of demographic variables such as race or gender as explanatory variables comes from the way that these variables confer access to resources or the degree to which they are indices of social inequality. Structural inequality is not just the direct experience of social hierarchy or the aggregation of adversity. It is the sum total of the ways that society actively fosters unequal experiences for some “lower status” members, through intentional government policies and societal customs actively enforced and historically entrenched. This wide-ranging set of experiences cannot be captured through one variable (e.g., exposure to poverty), through an aggregated set of adversity exposures, or by directly simulating inequality in a lab setting. Structural inequality encompasses not only the experiences that participants have but also the ones they are not able to have, and it includes experiences so subtle that learning they are happening sometimes requires the very education which is unequally provided.

“What this and related studies indicate is that structural inequality, the iterative and active process of maintaining social hierarchies through government actions and societal norms, is one mechanism through which gender as a social process becomes embodied leading to increased risk for negative health and well-being outcomes.”

In the recent study by Zugman et al., researchers observe that in countries with more indicators of gender equality, few differences in neural structure by gender were observed. When they do exist, they are suggestive of relatively positive outcomes for women relative to men (Zugman et al. 1). In contrast, in countries with more indicators of gender inequality, neural differences are stronger and show opposite patterns. This is one of only a few attempts to identify the impact of the aggregate exposure to social inequality on neural structure. In another recent paper, Weissman and colleagues demonstrated that differences in social safety nets across states within the United States moderate the impact of poverty on neural structure (9). These two papers demonstrate that indicators of inequality, measured at the level of political boundaries (e.g., nations or states), are associated with individual differences in brain structure. Given the distal relationship between country-level indicators of inequality and personal brain development, the mere fact that this association is identifiable is remarkable and points to the profoundly pervasive nature of gender inequality.

The implications of this development are multiple. First, this demonstrates the possibility that through these kinds of analyses, it may be possible to more fully identify the effect of structural inequality on biology, adding an additional source of information to an already substantial literature documenting links between person-level assessments of social inequality and neural structure. Second, these findings show that political structures likely constrain and shape biology, and in particular as observed here: brain structure. Individuals interested in understanding brain development or shaping a more just society must acknowledge that the laws which administer access to resources, health care, and education differentially across nations and across political boundaries within countries may shape the course, timing, and ultimate end point of the developing brain for some participants.

As the authors of this study point out, gender inequality in health is often explained as a consequence of differences in genetically determined biology or exposure to trauma. What this and related studies indicate is that structural inequality, the iterative and active process of maintaining social hierarchies through government actions and societal norms, is one mechanism through which gender as a social process becomes embodied leading to increased risk for negative health and well-being outcomes.
